# Feasibility of an incentivised exercise program to improve the health of physically inactive Australian hospital employees – the Fitbites pilot study

**DOI:** 10.1016/j.pmedr.2026.103404

**Published:** 2026-02-10

**Authors:** Christine M. Madronio, Andrea Tyler, Linda Stanbury, Gary K.K. Low, Deva R. Nirthanakumaran, Emmanuel Stamatakis, Kazuaki Negishi, Faraz Pathan

**Affiliations:** a Faculty of Medicine and Health, The University of Sydney, Sydney, NSW 2006, Australia; b Nepean Blue Mountains Family Metabolic Health Service, Nepean Hospital, Nepean Blue Mountains Local Health District, Derby St, Kingswood, NSW 2747, Australia; c The University of Sydney, Charles Perkins Centre, Sydney, NSW 2006, Australia; d Heart Failure Service, Nepean Hospital, Nepean Blue Mountains Local Health District, Derby St, Kingswood, NSW 2747, Australia; e Health Promotion Unit, Nepean Blue Mountains Local Health District, Lemongrove Campus, Gascoigne St, Penrith, NSW 2750, Australia; f Research Directorate, Nepean Hospital, Nepean Blue Mountains Local Health District, Derby St, Kingswood, NSW 2747, Australia; g Department of Cardiology, Nepean Hospital, Nepean Blue Mountains Local Health District, Derby St, Kingswood, NSW 2747, Australia; h Mackenzie Wearables Research Hub, Charles Perkins Centre, The University of Sydney, Sydney, NSW 2006, Australia; i School of Health Sciences, Faculty of Medicine and Health, The University of Sydney, Sydney, NSW 2006, Australia; j School of Clinical Medicine, University of New South Wales, Sydney, NSW 2052, Australia; k Department of Cardiology, Liverpool Hospital, Locked Bag 7103, Liverpool BC, NSW 1871, Australia; l The Ingham Institute for Applied Medical Research, PO Box 3151 (Westfields), Liverpool, NSW 2170, Australia; m Victor Chang Cardiac Research Institute, Lowy Packer Building, 405 Liverpool St, Darlinghurst, NSW 2010, Australia; n Heart Research Institute, 7 Eliza Street, Newtown, NSW 2042, Australia

**Keywords:** Exercise, Physical activity, Health workers, Workplace, Occupational, Incentive

## Abstract

**Objective:**

To investigate the feasibility of a 10-week incentivised exercise program (Fitbites) to improve the health of hospital employees.

**Methods:**

We recruited 20 employees who were deemed physically inactive from a busy, outer-metropolitan Australian hospital. They were invited to attend in-hours exercise sessions and redeem a healthy meal upon completing exercise as part of the Fitbites program, conducted between October and December of 2022. Feasibility and safety were assessed by evaluating attendance, meal redemption and completion of program. Pre- and post-program comparisons were made for body composition, functional capacity and blood parameters.

**Results:**

Among the 20 participants included in the study, 16 (80%) completed the program and were included in the analysis (2 were lost to follow-up, 2 withdrew). On average, employees attended 2.5 sessions per week. Most of the meal vouchers were redeemed (94.5%). All workdays and exercise sessions were well attended. There were decreases in body mass index (mean difference: −0.6 kg/m^2^) and fat mass index (−1.3 kg/m^2^), and increases in skeletal mass index (0.8 kg/m^2^) and 6-Minute Walk Test (45.9 m).

**Conclusions:**

The Fitbites program showed acceptable uptake and led to improvements in body composition and functional capacity. These findings inform future randomised trials in occupational settings.

## Highlights

• A dedicated exercise break for hospital employees improves physical activity.
• A healthy meal is a highly acceptable incentive for exercise participation.
• Our program increased women's involvement in resistance training exercise.
• Highly flexible health promotion programs are needed for hospital settings.

## 1 Introduction

The prevalence of overweight and obesity worldwide has increased dramatically in the last several decades (World Health Organization, 2025). Over half of adults were above the recommended body mass index (BMI), with 43% with overweight and 16% with obesity in 2022 (World Health Organization, 2025). Two-thirds of deaths related to high BMI are attributable to cardiovascular disease (G. B. D. Obesity Collaborators et al., 2017). Physical inactivity is associated with increased cardiovascular risk and as little as five minutes of stair climbing can improve cardiometabolic risk profile (Ahmadi et al., 2024; Koemel et al., 2025).

The workplace presents a good opportunity to make a difference to the health of employees. The World Health Organization's Global Plan of Action on Worker's Health encourages the promotion and prevention of non-communicable diseases in the workplace by advocating for physical activity among workers (World Health Organization, 2007).

Employers have trialled a wide variety of workplace health promotion programs (WHPP) that have led to improvements in the health of workers (Barr-Anderson et al., 2011; Bellicha et al., 2016; Commissaris et al., 2016; Reed et al., 2017; Rongen et al., 2013; White et al., 2016). These include stair-use and group step challenges (Bellicha et al., 2015; Bellicha et al., 2016; Duncan et al., 2019). Incentives such as text messages and provision of pedometers have also been effective strategies (Fournier et al., 2017; Hess et al., 2011; Thogersen-Ntoumani et al., 2015). WHPP have also demonstrated benefits to the employer, the average benefit-standardised return-on-investment was 200% (standard deviation [SD] 440%) for absenteeism benefits, 22% (SD 168%) for medical benefits, 246% (SD 557%) for presenteeism benefits and 174% (SD 438%) for both absenteeism and medical benefits (van Dongen et al., 2011).

However, authors of several systematic reviews on WHPP have commented on the lack of high-quality studies, the lack of reporting of various outcome measures and process evaluations, thus not allowing meta-analysis to be conducted to inform best evidence in this domain (Hutchinson and Wilson, 2012; Rongen et al., 2013; White et al., 2016; Wierenga et al., 2013). The recent review by Worley, et al., has echoed these sentiments and highlighted challenges for implementing such programs in hospital settings (Worley et al., 2022).

Unlike traditional workplaces with predictable hours, hospitals present unique challenges, including a high prevalence of shift workers who are at additional cardiovascular risk (Vestergaard et al., 2023). The emergency need for care of patients at the bedside, demanding workloads and poor self-care culture are barriers that make implementation of a single exercise break difficult (Pultz et al., 2023).

The aim of this pilot study was to investigate the feasibility of an incentivised 10-week exercise program for hospital employees. A secondary aim was to assess the pre-post pilot study intervention differences in biological and functional measurements.

## 2 Methods

### 2.1 Study design and setting

We conducted a single-site pilot study of a 10-week incentivised exercise program (Fitbites) targeted at employees of a busy outer-metropolitan Australian hospital during October to December of 2022. As the study was conducted towards the tail-end of the COVID-19 pandemic, a pragmatic sample of 20 participants was used to minimise disruption to the workforce and health service delivery at Nepean Hospital.

### 2.2 Participants and recruitment

Eligible individuals included employees and affiliates of the Nepean Blue Mountains Local Health District (NBMLHD) who were between 20 and 65 years old, deemed inactive (i.e., responded that they do not engage in ≥30 min of moderate physical activity at least three times a week, in our screening questionnaire), did not have a prior diagnosis of cardiovascular disease or other condition that will prohibit participation in physical activity, and who were able to attend exercise sessions for 10 weeks held at Nepean Hospital. The study was promoted through the staff newsletter, intranet, word of mouth and by direct email to department heads of the hospital. Expressions of interest were followed up by the research team who then contacted and explained the study to these individuals. Informed consent was obtained from potential participants prior to undergoing screening and inclusion in the study. They then underwent a three-stage screening process which included a questionnaire to determine eligibility, followed by assessment by an exercise physiologist to ascertain capacity for exercise and lastly, an exercise stress test supervised by a cardiologist. The study also recruited other hospital employees who volunteered to be fitness leaders, who led and ran the exercise sessions as part of the program. These individuals were also screened for eligibility. They underwent a clinical assessment by an exercise physiologist and were required to have a current First aid certificate. Fitness leader volunteers underwent a training program and were instructed on how to use an online rostering system. A primary and a back-up leader were rostered for each session.

An email was automatically sent to remind participants of their upcoming exercise session. Incentive text messages were sent to congratulate participants on their attendance or to remind them for non-attendance.

Healthy eating was encouraged and for every exercise session completed, participants were given a meal voucher that can be redeemed for a healthy salad, wrap or sandwich from the hospital cafe. The healthy meals met the requirements to be classified as an Everyday food in accordance with the Healthy Food and Drink in NSW Health Facilities Framework (NSW Ministry of Health, 2017). Participants received one voucher per exercise session completed, which they could use anytime within one week of date of issue.

**Fig. 1:**
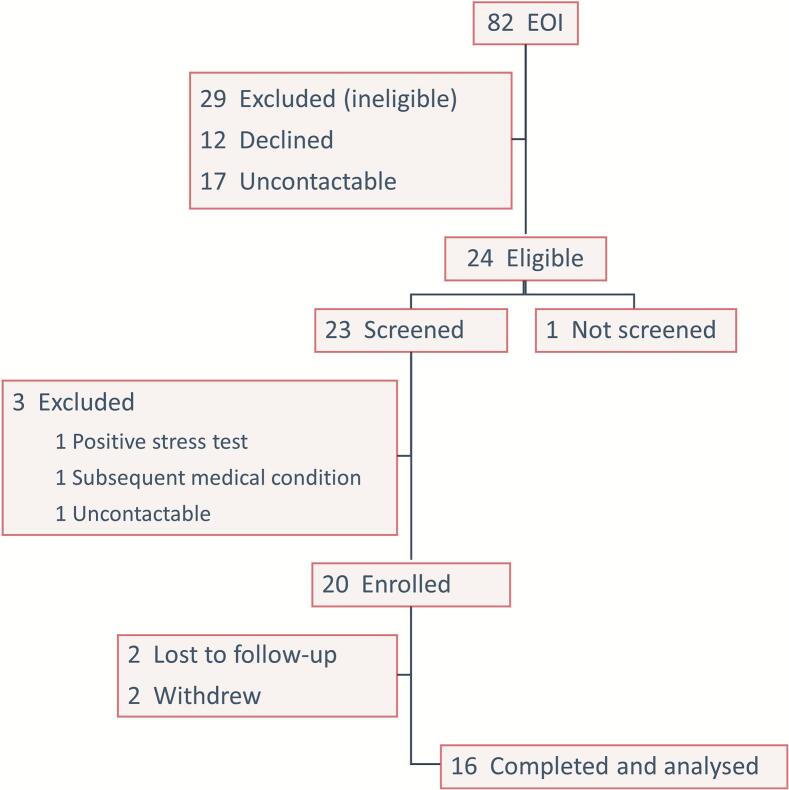
Flowchart of the selection process and exclusion criteria for hospital employees evaluated for Fitbites Pilot Study, October–December 2022, New South Wales, Australia. Abbreviation: EOI, expression of interest.

Expressions of interest to participate in the study were received from 82 employees (88% female) (Fig. 1). Twenty staff members were enrolled in the study. From the initial 20 participants, two were lost to follow-up and two withdrew. Reasons for withdrawal included: 1) an injury sustained at home which prevented participation in exercise, and 2) substantial leave during the program. A total of 16 participants (80%) completed the program and were included in the analysis (Fig. 1).

### 2.3 Fitbites program implementation

Participants were expected to engage in a 20-min supervised group exercise session at least three times a week for 10 consecutive weeks. They could attend as many sessions as they wanted and were given advice by an exercise physiologist regarding appropriate rest between sessions. Three sessions were offered on site and included walking (∼1 km route incorporating stairs, ramps and level ground), stairclimbing (with active rest) and resistance training. Resistance training was held at our outpatient gym using standard equipment. Additional information on the sessions is provided in **Supplemental method S1**. Participants were able to adjust the intensity of their resistance training, as needed. Exercise sessions were capped at 10 participants. To provide flexibility to shift workers, three sessions were offered per day (at 7:30 am, 12:00 pm, 4:30 pm), Monday to Friday. The three exercise types were distributed evenly throughout the week (**Supplemental table S2**). The same weekly schedule was followed for the duration of the program, with exercise type and fitness leader kept constant as much as possible. Participants were instructed to reserve their spot through an online booking system prior to attending a session.

Attendance at each exercise session was recorded. The number of vouchers redeemed for a healthy meal were also collected daily. Anthropometric, body composition, blood serum, vital signs and functional measurements were taken before and after the Fitbites program. Body composition was measured using the Bodystat® Quadscan 4000 (**Supplemental method S3**). The 6-Minute Walk Test was used to assess functional capacity. Blood samples were taken to assess participants' glucose, lipid and other cardiometabolic-related profiles. Participants also completed a program evaluation survey and other additional health questionnaires.

Key feasibility parameters were attendance, meal redemption, exercise preference, session attendance and weekday attendance. Furthermore, changes in exercise capacity, body composition and blood parameters were recorded.

A total of 17 volunteer fitness leaders were rostered to cover the same 15 sessions offered each week, as either primary or back-up leader. Each week, the program required a total of 15 h of rostered leader time (7.5 h each for a primary and a back-up leader). This translated to having 2 × 0.2 full-time equivalent employees working a standard 38-h week. Two participants had minor accidents due to a faulty weights machine resulting in short-term slight discomfort, both were able to continue with the program.

### 2.4 Statistical analysis

Descriptive statistics were reported as mean with standard deviation (SD) for continuous variables or as proportions expressed as percentages for categorical variables. Pattern of attendance was evaluated, and data is presented for the whole program and by week. Pre- and post-program biological and functional measurements were compared on a group and individual level. Group mean differences with confidence intervals were calculated. Analyses were performed using Microsoft® Excel, R Statistical Software (version 4.2.2) (R Core Team, 2022) and RStudio® (version 2022.07.2 + 576) (RStudio Team, 2022).

### 2.5 Ethical considerations

Study procedures were performed in accordance with institutional guidelines and relevant legislations. Ethical approval was obtained from the Nepean Blue Mountains Local Health District (NBMLHD) Human Research Ethics Committee (2019/ETH12829, 20 December 2019). The privacy rights of participants were observed and written, informed consent was obtained from all participants. Participants were informed that they could withdraw from the study at any time without consequences.

## 3 Results

### 3.1 Baseline characteristics

Participants were mostly female (14/16) and from various occupations within the hospital (clinicians = 7, managers = 2, technical/administrative/other staff = 7). The mean age was 48 ± 11 years. Participants ranged from normal weight (38%), to those with overweight (31%) and obesity (31%). Blood Pressure, Cholesterol and glucose were within normal ranges (Table 1**)**.

**Table 1:** Baseline demographic and metabolic characteristics of hospital employees who participated in the Fitbites program (*N* = 16), October–December 2022, New South Wales, Australia.

Characteristic	Mean ± SD a
Female, N (%)	14 (88)
Age (years), median (IQR)	51 (45, 55)
Body mass index, N (%)	
Normal weight	6 (38)
Overweight	5 (31)
Obese	5 (31)
Heart rate (bpm)	70 ± 9
Blood pressure (mmHg)	
Systolic	122 ± 12
Diastolic	80 ± 8
Cholesterol (mmol/L)	5.2 ± 1.1
Glucose (mmol/L)	5.2 ± 0.5

Abbreviations: IQR, interquartile range; SD, standard deviation.

a Data presented as mean ± SD, unless otherwise specified.

### 3.2 Feasibility - attendance, exercise preference and meal redemption

The 10-week program ran between October and December 2022, following the weekly schedule as shown in **Supplemental table S2**.

**Fig. 2:**
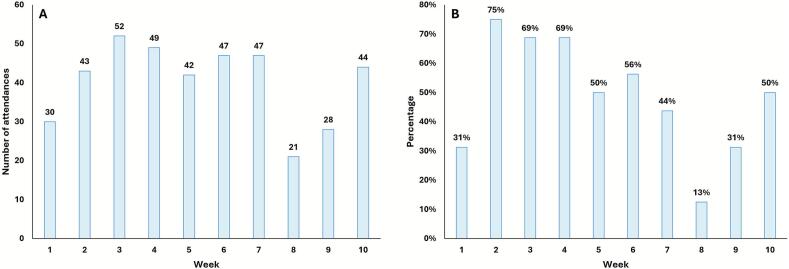
Patterns of attendance for hospital employees in the Fitbites Pilot Study, October–December 2022, New South Wales, Australia. Panel A. Number of attendances, by week of the program. Panel B. Percentage of hospital employees in Fitbites Program meeting the exercise target of ≥3 sessions per week, by week of the program.

As seen in Fig. 2**, Panel A**, attendance gradually increased from the start of the program and reached its peak in Week 3. Week 8 had the lowest attendance, increasing to earlier levels towards the end of the program. Participants were asked to attend at least three exercise sessions per week over 10 weeks. In Weeks 2, 3, 4 and 6, more than half of participants met their weekly target (Fig. 2**, Panel B**). In contrast, Week 8 only had two out of 16 participants meet their weekly target. On average, each participant attended 2.5 sessions per week.

**Fig. 3:**
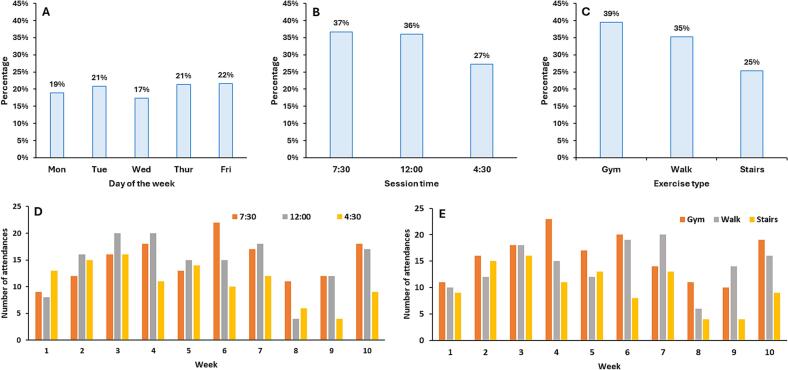
Patterns of participation for hospital employees in the Fitbites Pilot Study, October–December 2022, New South Wales, Australia. Percentage of participants attending sessions, by day of the week (Panel A), by session time (Panel B) and by exercise type (C) for the whole duration of the program. Number of attendances by session time (Panel D) and exercise type (Panel E) for each week of the program.

Overall, there were no clear trends regarding participants' preferred day of the week for exercising (Fig. 3**, Panel A**). Regarding session time, it appears that the 7:30 am and 12:00 pm sessions were preferred over the 4:30 pm session (Fig. 3**, Panel B and D**).

For exercise type, the gym sessions had the highest overall attendance (*n* = 159), followed by walking (*n* = 142) and stairclimbing (*n* = 102) (Fig. 3**, Panel C and E**).

Over the 10 weeks, a total of 403 meal vouchers were given out to participants, of which 94.5% (*n* = 381) were redeemed.

### 3.3 Health outcomes

**Fig. 4:**
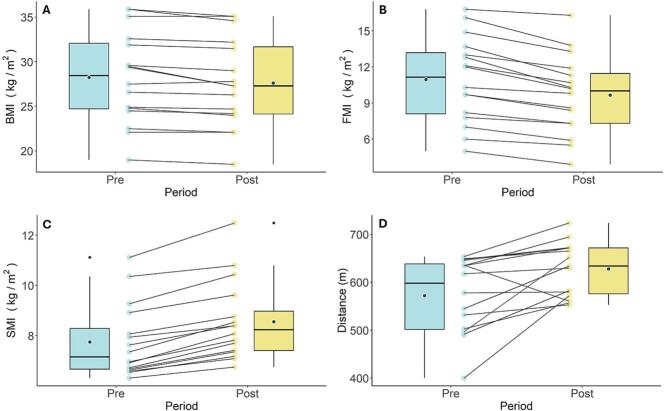
Plots showing changes in Body Composition and Exercise Capacity for hospital employees in Fitbites Pilot Study, October–December 2022, New South Wales, Australia. BMI (Panel A), FMI (Panel B), SMI (Panel C) and 6MWT distance (Panel D) pre- and post-intervention. Abbreviations: BMI, body mass index; FMI, fat mass index; SMI, skeletal mass index; 6MWT, 6-Minute Walk Test.

Table 2 summarises pre- and post-intervention changes. Improvements were observed in participants' body composition and functional capacity. Body mass index and fat mass index decreased compared to pre-program levels (Fig. 4**, Panels A and B**), with a mean difference of 0.6 kg/m^2^ and 1.3 kg/m^2^, respectively (Table 2). On the other hand, skeletal mass index increased compared to pre-program levels with a median difference of 0.8 kg/m^2^ (Fig. 4**, Panel C, and** Table 2). Participants' walking distance also increased by a median difference of 45.9 m after the program (Fig. 4**, Panel D, and** Table 2). There were no major differences for blood pressure, cholesterol, triglycerides, glucose and haemoglobin A1c (HbA1c). However, mean values before and after the program were within normal reference ranges (Table 2).

**Table 2:** Mean difference for blood pressure, body composition, 6-Minute Walk Test distance and metabolic parameters pre- and post-intervention for the hospital employees who participated in the Fitbites program (N = 16), October–December 2022, New South Wales, Australia.

Parameter	Mean			95% CI
	Pre	Post	Difference	Lower, upper
Blood pressure				
Systolic (mmHg)	122.1	124.5	2.4	−3.1, 7.9
Diastolic (mmHg)	80.4	79.5	−0.9	−4.1, 2.2
Body mass index (kg/m^2^)	28.2	27.6	−0.6	−1.0, −0.3
Fat mass index (kg/m^2^)	10.9	9.7	−1.3	−1.6, −0.9
Skeletal muscle index (kg/m^2^) a	7.2 (6.7,8.3)	8.2 (7.4,9.0)	0.8	
6MWT distance (m) a	598 (502,638)	634 (576,672)	45.9	
Cholesterol (mmol/L)	5.2	4.9	−0.3	−0.6, 0.1
Triglycerides (mmol/L)	1.2	1.3	0.1	−0.1, 0.3
Glucose (mmol/L)	5.2	5.1	−0.1	−0.3, 0.1
HbA1c (%)	5.5	5.4	−0.1	−0.2, 0.0

Abbreviations: CI, confidence interval; 6MWT, 6-min walk test; HbA1c, haemoglobinA1c.

a Median (interquartile range).

## 4 Discussion

We have demonstrated the feasibility of running a 10-week, incentivised exercise program for employees in a busy hospital setting. The Fitbites program incorporated supervised, group-based exercise sessions led by staff who became fitness leaders, with participation reinforced by a free healthy meal and text-based incentive system.

In a busy public hospital setting, it showed feasibility with modest uptake (average attendance of 2.5 sessions per week) and improvements in participants' body composition and functional capacity. Barriers such as shift work were addressed using three sessions per day to maximise opportunities to exercise. However, the average attendance of 2.5 sessions per week demonstrates the challenges of implementing protected exercise time within a very busy public hospital service.

It is well-known that more males than females participate in exercise science research (Nuzzo and Deaner, 2023). In our study, the level of initial interest and eventual participation in the study were mostly from female employees. This reflects the Australian health workforce, which is predominantly female (74%) (Australian Institute of Health and Welfare, 2024). We had a broad range of occupations within the hospital represented in this study including those engaged in shift work and on a regular work schedule. The difficulty of recruiting shift workers and the inclusion of other types of hospital employees besides nurses in workplace programs is an ongoing concern (Flahr et al., 2018; Worley et al., 2022). We recognise that some employees may be on their feet during the day, however, these employees by virtue of selection criteria identified themselves as inactive. Healthcare workers represent a unique population as they face additional metabolic stressors such as shift work and mental stress.

The use of a healthy meal incentive for exercising, was well received by participants, with 94.5% of meal vouchers being redeemed. This is consistent with the findings of other studies which reported the positive impact of incentives in their programs (Faghri and Li, 2014; Fournier et al., 2017; Hess et al., 2011; Thogersen-Ntoumani et al., 2015).

We were able to observe improvements in body composition and functional capacity after 10 weeks of exercise. A meta-analysis on the impact of workplace physical activity interventions on working-age women reported a significant reduction in BMI of 0.35 kg/m^2^ [95% CI 0.62, 0.07] (Reed et al., 2017). Our study showed a larger reduction in BMI (0.63 kg/m^2^ [1.0, 0.25]).

Our findings are in contrast with a previous review looking specifically at hospital-based interventions which reported non-significant reductions in BMI (Worley et al., 2022). Using body composition analysis, our study was able to unmask a more telling and important change occurring after the intervention, i.e., a more pronounced reduction in participants' fat mass index and a corresponding increase in their skeletal muscle index.

Consistent with the current literature (Pieper et al., 2019; Proper et al., 2003), our study has demonstrated the feasibility of conducting an exercise program in a busy hospital setting with the potential to improve the health of employees. The study also highlights the acceptability of strength training in a cohort that is predominantly female with a median age of 51 years. To improve attendance, more work needs to be done to “protect” employee exercise time. The use of a healthy meal as an incentive for exercising was widely accepted by participants in this study. This strategy is important, as it incentivises participants to attend an exercise session. Additionally, it gives us the opportunity to have an impact on a third of their daily dietary consumption.

The lack of preferred days and times for exercising suggest that participants needed the flexibility of availability of sessions, considering the nature of shift work and unpredictable workloads each day. Specific recruitment strategies and better alignment of exercise sessions with work patterns may be needed to increase participation in future studies.

There are limitations in this study. As this is a pilot study, the efficacy of the program could not be established despite the encouraging preliminary findings. Performing a larger, randomised control trial with a longer follow-up period can inform us of the effectiveness of the program but also show if health improvements are sustained over time. Given the small sample size of the study, there may also be potential for selection bias. Participants in the study were mostly female, and this may have had an impact on findings. Additionally, we did not strictly monitor changes in participants' daily physical activities and other health-related behaviours outside of the Fitbites program. Measuring changes in these areas during participation in the program would be very useful, as well as looking at the corresponding flow on effects on members of participants' immediate household. Finally, scalability of such an intervention with fitness leaders (calculated to be 0.4 full time equivalent), impact of meal incentives on hospital budgets and on service delivery needs to be evaluated in a larger implementation study. Indeed, any intervention at scale would be better served by dedicated staff delivering this intervention.

Nevertheless, this study provides valuable information on the feasibility of health promotion programs in hospital settings where data is currently limited. Our study makes use of a unique incentive to promote exercise participation, i.e., a healthy meal. This effectively ‘doubles’ the effect of the intervention, impacting employees' physical activity levels, as well as a third of their daily food intake. We were also able to recruit employees from a broad section of departments, incorporating different workloads and shift patterns. By including bioelectrical impedance analysis, we were able to report on more subtle body composition changes not always examined in previous studies.

## 5 Conclusions

The 10-week, incentivised exercise program during working hours in a busy hospital environment, showed moderate uptake, based on attendance patterns and led to improvements in body composition and functional capacity. These findings inform the design and implementation of future randomised trials in occupational settings.

## Credit author statement

• All authors listed provided assistance in executing this pilot study

1) Christine M Madronio: PhD Student and 1st author- ran and implemented entire Study
2) Andrea Tyler: Exercise physiologist, coordinated baseline, F/U assessment and interventional program- implementation
3) Linda Stanbury: Director of Health promotion- helped conceptualise and implement study
4) Gary KK Low: Statistician- provided statistical insight in analysis
5) Deva R Nirthanakumaran: Cardiologist, supervised baseline stress assessment and supervised exercise classes
6) Emmanuel Stamatakis: Professor of Physical activity and population health- Helped conceptualisation and editing of manuscript
7) Kazuaki Negishi: Professor of Medicine- helped with conceptualisation, Christines primary PhD supervisor
8) Faraz Pathan: Senior Author- helped conceptualisation, funding, implementation and writing of manuscript- Christine's secondary PhD supervisor

## Declaration of generative AI and AI-assisted technologies in the manuscript preparation process

The authors did not use generative AI or AI-assisted technologies in the preparation and writing of this work.

## D*isclosures

Faraz Pathan is supported by the New South Wales Health Cardiovascular Early-Mid Career Researcher Grant from the Office of Health and Medical Research. Christine Madronio was supported by a postgraduate research scholarship from the above grant received by Faraz Pathan. Emmanuel Stamatakis is a paid consultant and holds equity in Compliment 1, a US-based company whose services/products involve physical activity and exercise programs.

## CRediT authorship contribution statement

**Christine M. Madronio:** Writing – original draft, Methodology, Investigation, Formal analysis, Data curation, Conceptualization. **Andrea Tyler:** Project administration, Methodology, Conceptualization. **Linda Stanbury:** Project administration, Methodology, Funding acquisition, Data curation, Conceptualization. **Gary K.K. Low:** Methodology. **Deva R. Nirthanakumaran:** Project administration, Methodology. **Emmanuel Stamatakis:** Writing – review & editing, Methodology, Conceptualization. **Kazuaki Negishi:** Writing – review & editing, Conceptualization. **Faraz Pathan:** Writing – review & editing, Supervision, Resources, Methodology, Investigation, Funding acquisition, Conceptualization.

## Funding sources

This work was supported by the Health Promotion Unit, NBMLHD The funder had no involvement in the study design, collection, analysis and interpretation of data, writing and preparation of this work.

## Declaration of competing interest

The authors declare the following financial interests/personal relationships which may be considered as potential competing interests: Emmanuel Stamatakis is a paid consultant and holds equity in Compliment 1, a US-based company whose services/products involve physical activity and exercise programs. The remaining authors declare that they have no known competing financial interests or personal relationships that could have appeared to influence the work reported in this paper.

## Data Availability

Data will be made available from the corresponding author upon request, in accordance with ethical approvals.

## Appendix A Supplementary data

Supplementary material- on exercise sessions, body composition and weekly schedule Image 1
