# Supplementary material for: Feasibility of an incentivised exercise program to improve the health of physically inactive Australian hospital employees – the Fitbites pilot study
Source: Prev Med Rep. 2026 Feb 10;63:103404. doi: 10.1016/j.pmedr.2026.103404 (PMC12925218; doi:10.1016/j.pmedr.2026.103404)
Supplement: Supplementary file 1 — Supplementary material- on exercise sessions, body composition and weekly schedule [file mmc1.docx]

# Supplemental Material

## Supplemental methods

Supplemental method S1. Additional information on the exercise sessions, page 2

Supplemental method S3. Additional information on the body composition assessment, page 3

## Supplemental tables

Supplemental table S2. Weekly schedule of exercise sessions, page 4

## Supplemental method S1. Additional information on the exercise sessions

There were three types of sessions which ran for 20 minutes:

Walking:

Two walking routes were established to accommodate for wet/hot weather conditions. The routes covered approximately ~1,000 m and included level ground, ramps and stairs, in and around the hospital campus. The distance walked in each session may differ depending on the level of fitness of the participants in attendance.

### Stairclimbing (with active rest):

This session was conducted in an undercover five-level multi-storey carpark within the hospital campus. Participants climbed up to the top level and down to the ground level, had active rest, and repeated climbing up and down again. Active rest included one set of 20 jumping jacks, high-knees and body-weight jump squats. Modifications were made to the exercises depending on the level of fitness of the participants in attendance.

### Resistance training:

This session was conducted in the outpatient rehabilitation gym of the hospital and used standard gym equipment. Fifteen exercise stations were set up, with participants exercising at each station for one minute followed by a 15-second rest period and then moving on to the next station. The following exercises were included: wall push-ups, supine bridge, bent over single-arm row, leg press, chest press, crunches, side raise, step up, tricep kickback, hip abduction, lat pulldown, calf raises, bicep curls, incline superman and squats.

### Supplemental method S3. Additional information on the body composition assessment

Body composition was measured by bioelectrical impedance analysis (BIA) using the Bodystat® Quadscan 4000. This device applies a small electrical current to the body to measure the resistance of body tissues to the flow of the current using different frequencies.

Prior to their assessment, participants were instructed to avoid drinking or eating 4-5 hours before the appointment. During the assessment, participants lay on the bed and electrodes were placed on their hand and foot. Using the individual’s body weight, height, age and sex, the device was used to calculate body composition parameters, including skeletal muscle mass and fat mass.

## Supplemental table S2. Weekly schedule of exercise sessions

| **Time** | **Monday** | **Tuesday** | **Wednesday** | **Thursday** | **Friday** |
| --- | --- | --- | --- | --- | --- |
| 7:30 am | Gym | Walk | Gym | Gym | Walk |
| 12:00 pm | Walk | Stairs | Stairs | Walk | Gym |
| 4:30 pm | Stairs | Gym | Walk | Stairs | Stairs |
